# Factors limiting glaucoma care among glaucoma patients in Nigeria: A scoping review

**DOI:** 10.1371/journal.pgph.0002488

**Published:** 2024-01-26

**Authors:** Osamudiamen Cyril Obasuyi, Olabisi Olayemi Yeye-Agba, Oghenevwaire Joyce Ofuadarho

**Affiliations:** 1 Department of Ophthalmology, Irrua Specialist Teaching Hospital, Irrua, Edo State, Nigeria; 2 Department of Ophthalmology, Federal Teaching Hospital, Lokoja, Kogi State, Nigeria; PLOS: Public Library of Science, UNITED STATES

## Abstract

Glaucoma currently accounts for 11% of irreversible visual loss worldwide. Due to many factors, patients do not access the glaucoma care pathway and present late with poor vision, while many are undiagnosed or untreated. These factors may be personal-level dispositions or Institutional-level dispositions, limiting the awareness, diagnosis, and treatment of glaucoma or adherence to medications or follow-up clinic visits. This scoping review followed the JBI methodology for scoping reviews and was pre-registered on the open science platform (https://osf.io/wqx57/?view_only=727eb6c803764509a2809e5d0794e214). The PUBMED, EMBASE, WEB OF SCIENCE, AJOL, and GOOGLE SCHOLAR databases were systematically searched for studies published in English between 1990 and June 2023. Data were extracted and analysed along a conceptualised framework of factors limiting access to glaucoma care in Nigeria. Of the 336 records retrieved, 13 studies were included in this scoping review. These included one (1) mixed method (quantitative/qualitative) study, three qualitative studies, and nine quantitative studies spanning 2008–2022 covering eight states and 2,643 sampled respondents. Nine studies reported personal-level dispositions limiting glaucoma care, including low levels of education, unemployment, gender, living distance from the hospital, cost of care, and faith/religion. Four reported institutional-level dispositions, including the lack of proper equipment and expertise to diagnose or manage glaucoma. The factors limiting Glaucoma care in Nigeria are varied and may act alone or combined with other elements to determine the awareness or knowledge of glaucoma, uptake of glaucoma surgery, medication adherence, or clinic follow-up. While most of these factors limiting glaucoma care in Nigeria may be amenable to policy, a bottom-up approach is needed to improve the community’s awareness and uptake of glaucoma services. A shift from the over-dependence and reliance on tertiary hospitals, which are often far away from the people who need them, is required to bridge the information and service gap currently being witnessed.

## Introduction

Glaucoma is the “silent thief of sight” because it remains asymptomatic mainly throughout a significant part of the natural course of the disease, and patients only begin noticing symptoms at an advanced stage. Globally, glaucoma is responsible for about 11% of irreversible visual loss in adults 50 years or older [1, 2]. In Nigeria, the prevalence of glaucoma is 5%, and 94% of people with glaucoma are either undiagnosed or untreated. Most patients present blind to the clinic [3–5], and despite the high prevalence of glaucoma blindness, awareness about the disease is still poor [6, 7]. The natural course of the disease provides an avenue for preventing glaucoma blindness by improving the awareness of glaucoma, early detection of glaucoma, and treatment adherence [8–10].

**Fig 1:**
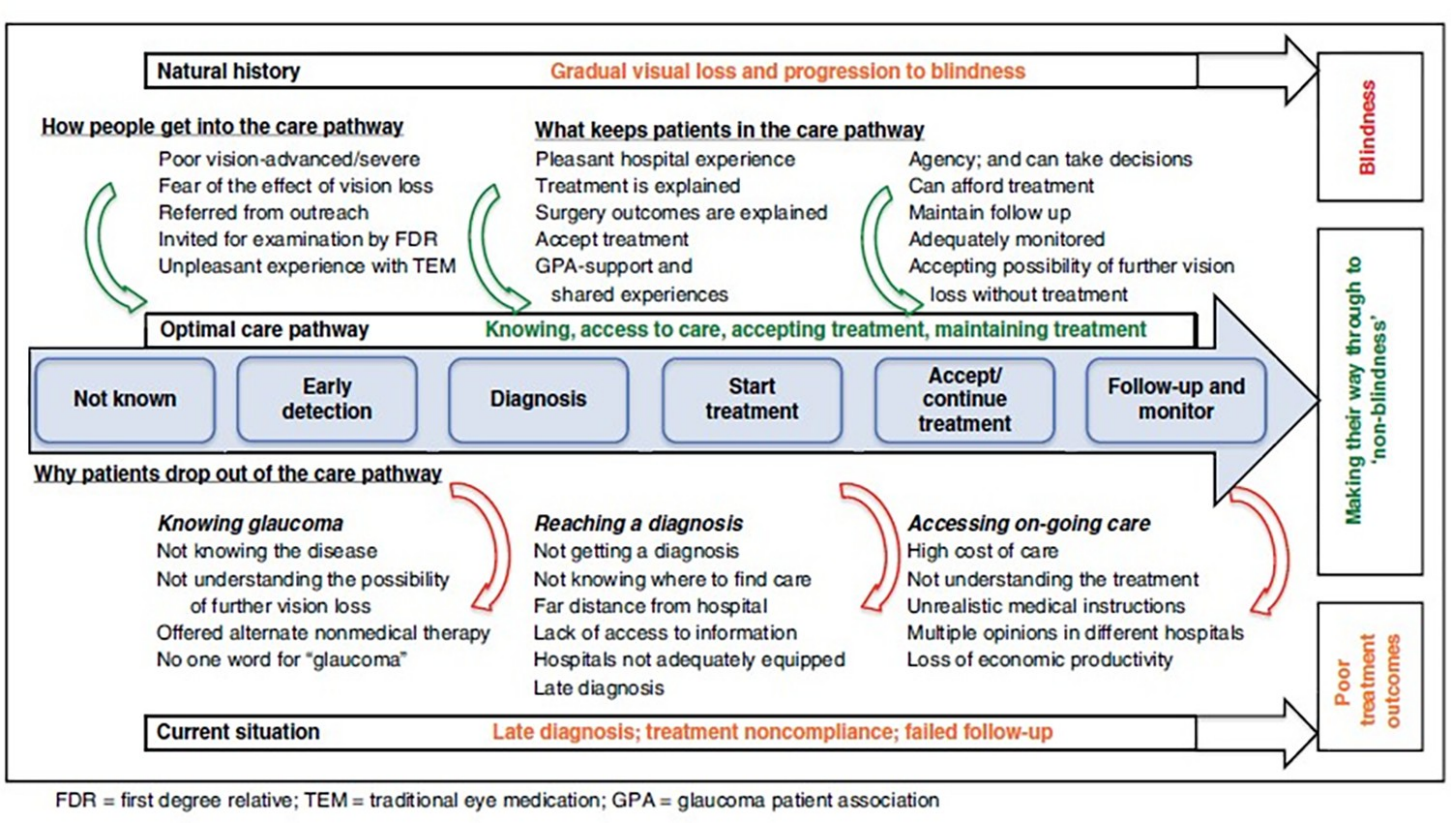
The glaucoma care pathway. FDR = first degree relative; TEM = traditional eye medication; GPA = glaucoma patient association.

As conceptualised by Kyari and colleagues, the Glaucoma care pathway describes the optimum pathway patients follow in pursuing glaucoma care to “non-blindness” [11]. (Fig 1) by providing avenues or access to information about glaucoma, available resources for the diagnosis and treatment of glaucoma, and support for adherence to treatment and hospital visits.

Unfortunately, the glaucoma care pathway may be affected by various factors which prevent optimum care, leading to blindness from glaucoma. Factors peculiar to the individual (individual-level dispositions) like age, gender, socio-economic status, occupation, level of education, and place of residence have been identified as essential social determinants of blindness from glaucoma [11–14]. These factors may limit the awareness of the disease, access to proper diagnosis and available resources to manage or treat glaucoma [15, 16]. Furthermore, systemic factors (Institutional-level dispositions) like poor healthcare funding, inadequate healthcare worker motivation, and lack of trained personnel may prevent the proper diagnosis or treatment of people with glaucoma and limit the ability of the patient to adhere to treatment [11, 17–19]. With the high prevalence of glaucoma, rates of undiagnosed cases, and poor vision at presentation in Nigeria, identifying the factors responsible for the poor utilisation of available resources for detecting and treating glaucoma becomes very important.

While many reports exist about the barriers to either treatment or adherence to glaucoma care, this scoping review aimed to identify how these factors limit the utilisation of glaucoma care in Nigeria by mapping the available evidence on the factors limiting glaucoma awareness, diagnosis, treatment, adherence, and follow-up of glaucoma among glaucoma patients in Nigeria.

### Review question

1. What are the factors limiting glaucoma care among glaucoma patients in Nigeria?
2. How do these factors predict the utilisation of glaucoma care along the care pathway among glaucoma patients in low-resource communities like Nigeria?

## Methods

### Protocol

A preliminary search of MEDLINE, the Cochrane Database of Systematic Reviews, and *JBI Evidence Synthesis* was conducted, and no current or underway systematic reviews or scoping reviews on the topic were identified. This scoping review followed the JBI methodology for scoping reviews [20] and was pre-registered on the open science framework. Pre-registration details can be found here: (https://osf.io/wqx57/?view_only=727eb6c803764509a2809e5d0794e214).

### Conceptual framework

**Fig 2:**
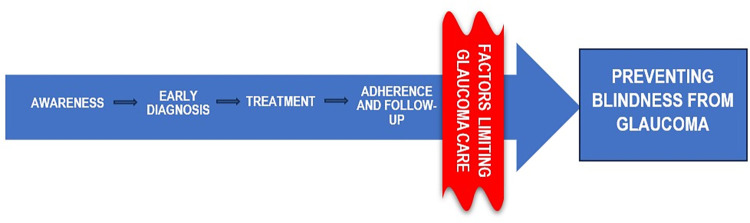
Conceptual framework on the factors limiting access to glaucoma care.

We conceptualised that a constellation of factors which could be due to personal-level dispositions stemming from the social determinants of blindness [12–14] or Institutional-level dispositions [17–19] act together rather than solitarily along the glaucoma care pathway preventing the receipt of optimum glaucoma care (Fig 2). In this conceptual model, we imagine that these factors associated with glaucoma care act at different points of the pathway, preventing the achievement of “non-blindness”.

### Eligibility criteria

The eligibility criteria for this review were based on the Participants, Concept, and Context (PCC) framework.

#### Participants

All studies of Nigerian patients diagnosed with glaucoma and healthcare workers managing glaucoma were included in this review. Studies of Nigerian patients diagnosed with any other eye disease were excluded.

#### Concept

All studies of patients diagnosed with glaucoma or healthcare workers managing glaucoma, which discussed some barriers to access care, were also included. These barriers may address awareness, diagnosis, treatment, adherence, or follow-up specifically or in combination.

#### Context

This review considered only studies done in Nigeria and among Nigerian patients diagnosed with glaucoma and Nigerian healthcare workers.

### Information sources

This scoping review considered observational studies, including prospective and retrospective cohort studies and analytical cross-sectional studies. This review also considered descriptive observational study designs, including case series and descriptive cross-sectional studies for inclusion. Qualitative studies on qualitative data describing the experiences of glaucoma patients, caregivers, and health workers were also included. Text and opinion papers were not considered for inclusion in this scoping review.

### Search strategy

The search strategy was designed to locate all published studies meeting the eligibility criteria. An initial limited search of MEDLINE and PUBMED was undertaken to identify articles on the topic on June 4, 2023. The text words contained in the titles and abstracts of relevant articles and the index terms used to describe the articles were used to develop a complete search strategy for (*PUBMED*, *EMBASE*, *WEB OF SCIENCE*, *SCOPUS*, *and AJOL) see* S1 Fig. Database search was carried out between 7th and 12th June 2023. The search strategy, including all identified keywords and index terms, was adapted for each included database and information source. The reference list of all included sources of evidence was screened for additional studies.

Only studies published in English were included in the search. Furthermore, the search period consisted of all studies published between 1990 and June 2023 to enable the inclusion of all relevant articles on the subject.

### Study/Source of evidence selection

Following the search, all identified citations were collated and uploaded to Rayyan(www.rayyan.ai), and duplicates were automatically removed. Titles and abstracts were screened independently by OCO, OOY, and OJO against the inclusion criteria for the review. After reviewing titles and abstracts, OCO and OJO independently assessed the full text of selected citations in detail against the inclusion criteria. Reasons for excluding sources of evidence in the full text that do not meet the inclusion criteria were recorded and reported in the scoping review. Any disagreements between the reviewers at each stage of the selection process were resolved through discussion or with an additional reviewer/s. The search results and the study inclusion process are reported and presented in the Preferred Reporting Items for Systematic Reviews and Meta-analyses extension for scoping review (PRISMA-ScR) flow diagram [21, 22].

### Data extraction

Data were independently extracted from papers included in the scoping review by OCO, OJO and OOY using a data extraction tool developed by the reviewers. The data extracted included specific details about the participants, concept, context, study methods, and key findings relevant to the review question/s.

Any reviewer disagreements were resolved through discussion or with an additional reviewer/s. Authors of papers were contacted to request missing or other data, where required.

### Methodological quality appraisal

This scoping review did not appraise any methodological quality or risk of bias in line with the guidance on scoping reviews [23].

### Synthesis

The synthesis involved a narrative analysis of the presented results from the studies based on the conceptual framework of the factors limiting glaucoma care among patients in Nigeria. Results were independently synthesised by OCO and OOY and compared before analysis. Any differences in synthesis were resolved by discussion or with an additional reviewer.

## Results

### Literature search

**Fig 3:**
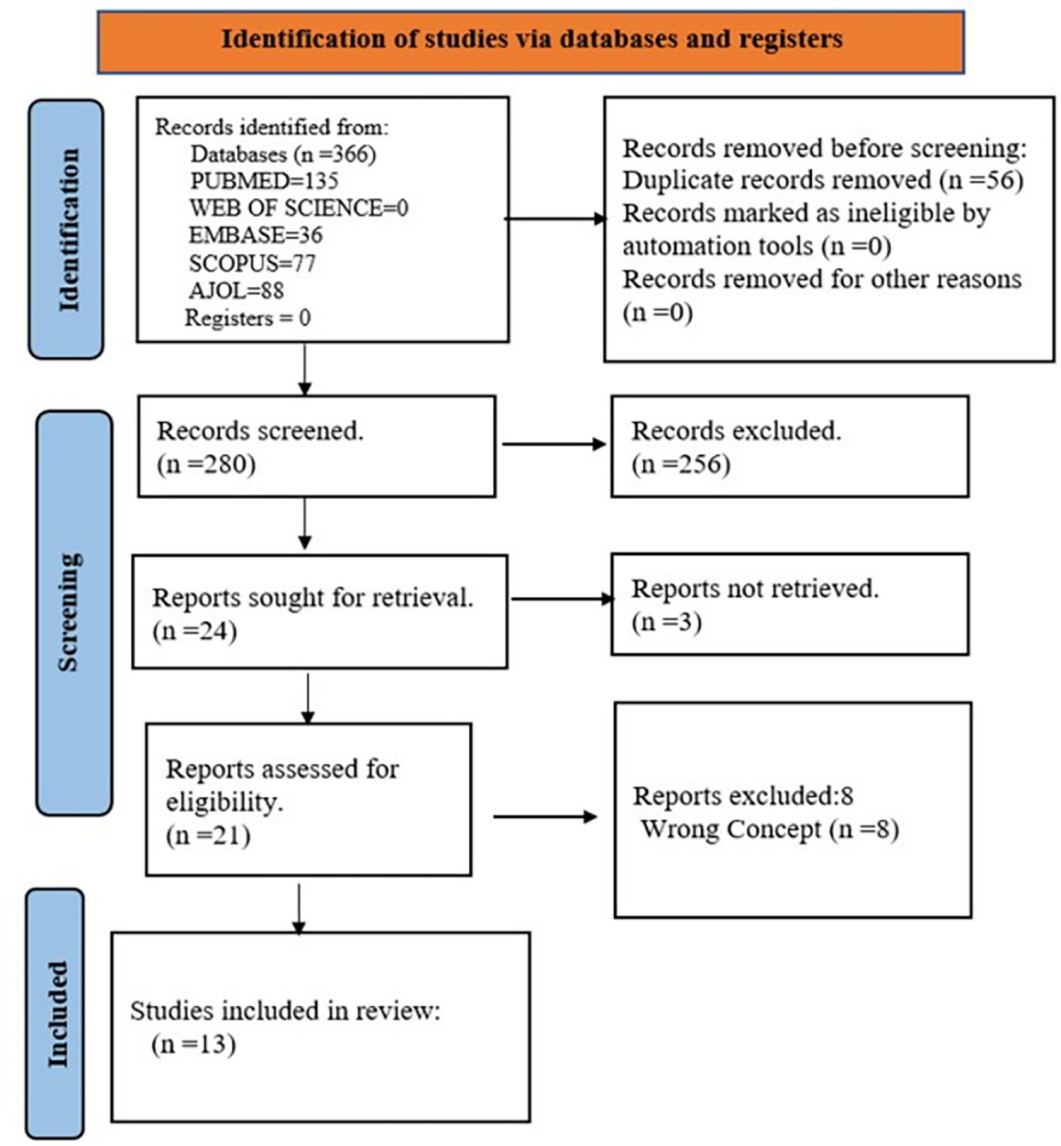
PRISMA flow diagram.

Three hundred thirty-six (336) records were retrieved on a literature search (Fig 3), including 56 duplicates. Two hundred eighty-one (280) records’ abstracts were screened, and 24 full texts were sought for retrieval. The full texts of three (3) reports were not retrieved and, hence, were not included in the final full-text assessments. Following full-text reviews, eight (8) studies were excluded because they failed to meet the eligibility criteria (wrong concept). Thirteen (13) reports were included in the final analysis and data synthesis. Of these, there was one (1) mixed method (quantitative/qualitative) study [15], three (3) qualitative reports [3, 11, 24], and nine (9) quantitative studies [16, 25–32].

### Study characteristics

Studies included in this report spanned the periods between 2008 and 2022, covering eight states, including Oyo State [15, 29, 31], Lagos [26, 33], Osun [27], Bauchi [3, 28], Enugu [16, 25, 32], Benin [30], Kaduna and Abuja FCT [11] (**Table 1**). A total of 2,643 respondents were sampled in the included reports of this review, and their socio-demographic data is shown below in **Table 2**.

**Table 1:** Study characteristics.

AUTHOR	YEAR	JOURNAL	STUDY METHOD	SETTING	LOCATION	SAMPLE SIZE
NKIRU N. KIZOR-AKARAIWE	2018	OPHTHALMIC EPIDEMIOLOGY	ANALYTICAL CROSS-SECTIONAL STUDY	FREE EYE SCREENING	ENUGU	182
OLUSOLA OLAWOYE	2022	JOURNAL OF THE WACS	MIXED METHODS ANALYTICAL STUDY	COMMUNITY EYE OUTREACH	IBADAN	120
BOLA J. ADEKOYA	2013	MIDDLE EAST AFRICAN JOURNAL OF OPHTHALMOLOGY	DESCRIPTIVE CROSS-SECTIONAL STUDY	HOSPITAL-BASED STUDY	LAGOS	208
MICHAELINE A. ISAWUMI	2014	MIDDLE EAST AFRICAN JOURNAL OF OPHTHALMOLOGY	DESCRIPTIVE CROSS-SECTIONAL STUDY	COMMUNITY EYE OUTREACH	OSUN	259
NKECHI J. UCHE	2020	INTERNATIONAL OPHTHALMOLOGY	DESCRIPTIVE CROSS-SECTIONAL STUDY	HOSPITAL-BASED STUDY	ENUGU	120
MOHAMMED M. ABDULL	2015	BMC OPHTHALMOLOGY	ANALYTICAL CROSS-SECTIONAL STUDY	HOSPITAL-BASED STUDY	BAUCHI	131
RITA O. MOMOH	2018	OPHTHALMIC EPIDEMIOLOGY	DESCRIPTIVE CROSS-SECTIONAL STUDY	HOSPITAL-BASED	BENIN	348
OLUSOLA OLAWOYE	2021	BRITISH JOURNAL OF OPHTHALMOLOGY	PROSPECTIVE COHORT STUDY	HOSPITAL-BASED/COMMUNITY OUTREACH	IBADAN	243
ADEYINKA O. ASHAYE	2008	JOURNAL OF GLAUCOMA	DESCRIPTIVE CROSS-SECTIONAL STUDY	HOSPITAL-BASED	IBADAN	747
ONWUBIKO S. N	2019	INTERNATIONAL OPHTHALMOLOGY	DESCRIPTIVE CROSS-SECTIONAL STUDY	HOSPITAL-BASED	ENUGU	88
MOHAMMED M. ABDULL	2016	BMC OPHTHALMOLOGY	FOCUSSED GROUP DISCUSSIONS	HOSPITAL-BASED	BAUCHI	66
BOLA J. ADEKOYA	2015	NIGERIAN JOURNAL OF MEDICINE	IN-DEPTH INTERVIEWS	HOSPITAL-BASED	LAGOS	11
FATIMA KYARI	2016	GLOBAL HEALTH ACTION	1. FOCUSSED GROUP DISCUSSIONS 2. IN-DEPTH INTERVIEWS 3. DIRECT OBSERVATION 4. EXIT INTERVIEWS	1. HOSPITAL-BASED 2. COMMUNITY-BASED	ABUJA/FCT KADUNA	120

**Table 2:** Study demographics.

AUTHOR	MEAN AGE	MALE	FEMALE	EDUCATION	PREDOMINANT OCCUPATION	RESPONDENTS RESIDENCE
NKIRU N. KIZOR-AKARAIWE	50	66.50%	33.50%	1. PRY = 90.1%	SELF EMPLOYED	URBAN/RURAL MIX
OLUSOLA OLAWOYE	59.4	56.70%	43.30%	1.NONE = 19.2% 2. PRY/SEC = 58.3% 3. TERTIARY = 21.7%	NOT PROVIDED	URBAN/RURAL MIX
BOLA J. ADEKOYA	54.2	56.70%	43.3	1.NONE = 13.0% 2. PRY/SEC = 65.9% 3. TERTIARY = 21.1%	1. SELF EMPLOYED 2. CIVIL SERVANTS 3. PENSIONERS	URBAN
MICHAELINE A. ISAWUMI	49.7	38.60%	61.40%	NOT PROVIDED	1. PROFESSIONAL 2. SELF EMPLOYED	RURAL
NKECHI J. UCHE	61.44	62.50%	37.50%	1. NONE = 29.2% 2. PRY/SEC = 49.2% 3. TERTIARY = 21.7%	1. SELF EMPLOYED 2. CIVIL SERVANTS 3. PENSIONERS	URBAN
MOHAMMED M. ABDULL	52.8	62.00%	38.00%	1. LITERATE = 42.7% 2. ILLITERATE = 57.3	1. SELF EMPLOYED 2. CIVIL SERVANTS 3. PENSIONERS	URBAN/RURAL MIX
OLUSOLA OLAWOYE	A. 59.4 B. 54.9	56.70% 52.80%	43.30% 47.20%	1.NONE = 19.3%(M)/12.2%(F) 2. PRY/SEC = 58.8%(M)/33.3%(F) 3.TERTIARY = 21.9%(M)/54.5%(F)	NOT PROVIDED	URBAN/RURAL MIX
RITA O. MOMOH	52.7	54.00%	46.00%	NOT PROVIDED	1. PROFESSIONALS 2. CIVIL SERVANTS 3. SELF EMPLOYED 4. UNEMPLOYED	URBAN/RURAL MIX
ADEYINKA O. ASHAYE	51	58.90%	41.10%	NOT PROVIDED	1. SELF EMPLOYED 2. CIVIL SERVANTS 3. PENSIONERS	URBAN/RURAL MIX
ONWUBIKO S. N	42.2	52.30%	47.70%	TERTIARY	1. OPHTHALMOLOGISTS 2. DIPLOMATES 3. RESIDENTS	URBAN
MOHAMMED M. ABDULL	NOT PROVIDED	NOT PROVIDED	NOT PROVIDED	LOW	FARMERS	RURAL
BOLA J. ADEKOYA	NOT PROVIDED	54.50%	45.50%	TERTIARY	PROFESSIONAL	URBAN
FATIMA KYARI	NOT PROVIDED	50%	50%	NOT PROVIDED	1. SELF EMPLOYED 2. CIVIL/PUBLIC SERVANTS	URBAN/RURAL MIX

### Factors limiting glaucoma care

The factors limiting Glaucoma care are detailed in **Tables 3 and 4** below. Nine studies focussed on personal-level dispositions limiting glaucoma care. Of these nine studies, five focussed on awareness and knowledge [11, 15, 16, 27, 33], two focussed on factors impacting the diagnosis of glaucoma [11, 29], four on factors limiting glaucoma surgical uptake [3, 16, 27, 34]. In addition, three studies looked at patient adherence to medication [3, 15, 34], while four studied factors limiting clinic follow-up [15, 25, 30, 31]. No study reported personal-level dispositions restricting the uptake of medical glaucoma care.

**Table 3:** Personal-level dispositions limiting glaucoma care.

			TREATMENT		FOLLOW-UP	
AUTHOR	AWARENESS/KNOWLEDGE	DIAGNOSIS	MEDICAL	SURGICAL	MEDICATION	CLINIC VISITS
NKIRU N. KIZOR-AKARAIWE	Was not studied	Was not studied	Was not studied	Was not studied	Was not studied	Cost, distance, busy schedule, lack of information
OLUSOLA OLAWOYE	No/primary education, female gender, no previous eye check	Was not studied	Was not studied	Was not studied	Cost, the belief that medication was unnecessary	Faith/ religion, cost, no information about follow-up centres
BOLA J. ADEKOYA	No/primary education, No previous eye check	Was not studied	Was not studied	Fear, Cost, Faith/religion, No visual improvement, Negative publicity	Costs, availability of medications, presence of counterfeit drugs	Was not studied
MICHAELINE A. ISAWUMI	Younger age, Female gender, Unskilled professionals	Was not studied	Was not studied	No visual improvement	Was not studied	Was not studied
NKECHI J. UCHE	Older age, >10km from hospital, No education, Unemployed, Female gender	Was not studied	Was not studied	Fear, Cost, Faith/religion, No visual improvement	Was not studied	Was not studied
MOHAMMED M. ABDULL	Was not studied	Was not studied	Was not studied	Fear, Desire to continue medical treatment, cost, no time, need to consult family.	Forgetfulness, beliefs about medication/side effects/efficacy, poor information about medication use, costs, availability of medications	Was not studied
OLUSOLA OLAWOYE	Was not studied	Older age, No/primary education, distance from the hospital, Lack of previous glaucoma awareness, Lack of felt need, Cost	Was not studied	Was not studied	Was not studied	Was not studied
RITA O. MOMOH	Was not studied	Was not studied	Was not studied	Was not studied	Was not studied	Poor vision, the severity of glaucoma, distance from the hospital, younger age <40
ADEYINKA O. ASHAYE	Was not studied	Was not studied	Was not studied	Was not studied	Was not studied	Older age group, males, the severity of glaucoma, distance from the hospital, no previous family history of blindness, polydrug use, fear
FATIMA KYARI	The stigma around blindness, misconceptions about eye diseases, poor access to information about glaucoma	Lack of information about where to seek care, costs, lack of autonomy, and the distance to the hospital.	Costs	Was not studied	Was not studied	Was not studied

**Table 4:** Institutional-level dispositions limiting glaucoma care.

			TREATMENT		FOLLOW-UP	
AUTHOR	AWARENESS/KNOWLEDGE	DIAGNOSIS	MEDICAL	SURGICAL	MEDICATION	CLINIC VISITS
NKIRU N. KIZOR-AKARAIWE	Was not studied	Was not studied	Was not studied	Was not studied	Was not studied	Discourteous staff
ONWUBIKO S. N	Was not studied	Lack of functional equipment, costs, lack of expertise in early glaucoma diagnosis	Was not studied	Satisfaction with visual results, fear of complications, costs, length of post-op care	Was not studied	Was not studied
BOLA J. ADEKOYA	Was not studied	Lack of equipment, poor maintenance of available equipment	Was not studied	Surgeon’s reluctance to offer surgery.	Was not studied	Was not studied
FATIMA KYARI	Was not studied	Lack of proper referral system, lack of trained personnel	Was not studied	Was not studied	Was not studied	Was not studied

On the other hand, only four studies reported results regarding the institutional-level dispositions limiting the provision of glaucoma care. While three studies reported on the institutional factors limiting the diagnosis of glaucoma [11, 24, 32], two studies reported on glaucoma surgical uptake [24, 32], and one reported on clinic follow-up visits [25]. No study reported on the institutional-level dispositions affecting the medical treatment of glaucoma or the adherence to medical therapy.

Factors which limited the awareness of the disease or avenues to access care included low levels of education [15, 16, 26], no previous eye checks [15, 26], female gender [15, 16, 27], older age [16], leaving greater than 10km from the hospital [16], the stigma around blindness [11], poor access to glaucoma information [11] and unemployment [16]. Lack of information, lack of felt need, long distance to the hospital, and high costs [11, 29] limited access to proper glaucoma diagnosis. Furthermore, fear of the surgical procedure, faith/religious beliefs and a lack of visual improvement following surgery [3, 16, 26, 27] were the factors identified that limited the uptake of glaucoma surgeries. Adherence to medical treatment and clinic visits was hampered by costs of medications and transportation, faith/religious beliefs, lack of availability of drugs and the fear of medication side effects [3, 15, 25, 26, 30, 31]. In addition, the factors which were reported to limit the diagnosis of glaucoma included a Lack of functional equipment [24, 32], a lack of expertise in glaucoma diagnosis, high costs of accessing care [32] and a lack of a proper referral system [11]. The poor satisfaction with visual results following surgery, reluctance to offer surgery, the fear of complications, and the costs prevented the uptake of glaucoma surgeries [24, 32]. Discourteous staff were why patients were unwilling to return for clinic visits [25].

**Fig 4:**
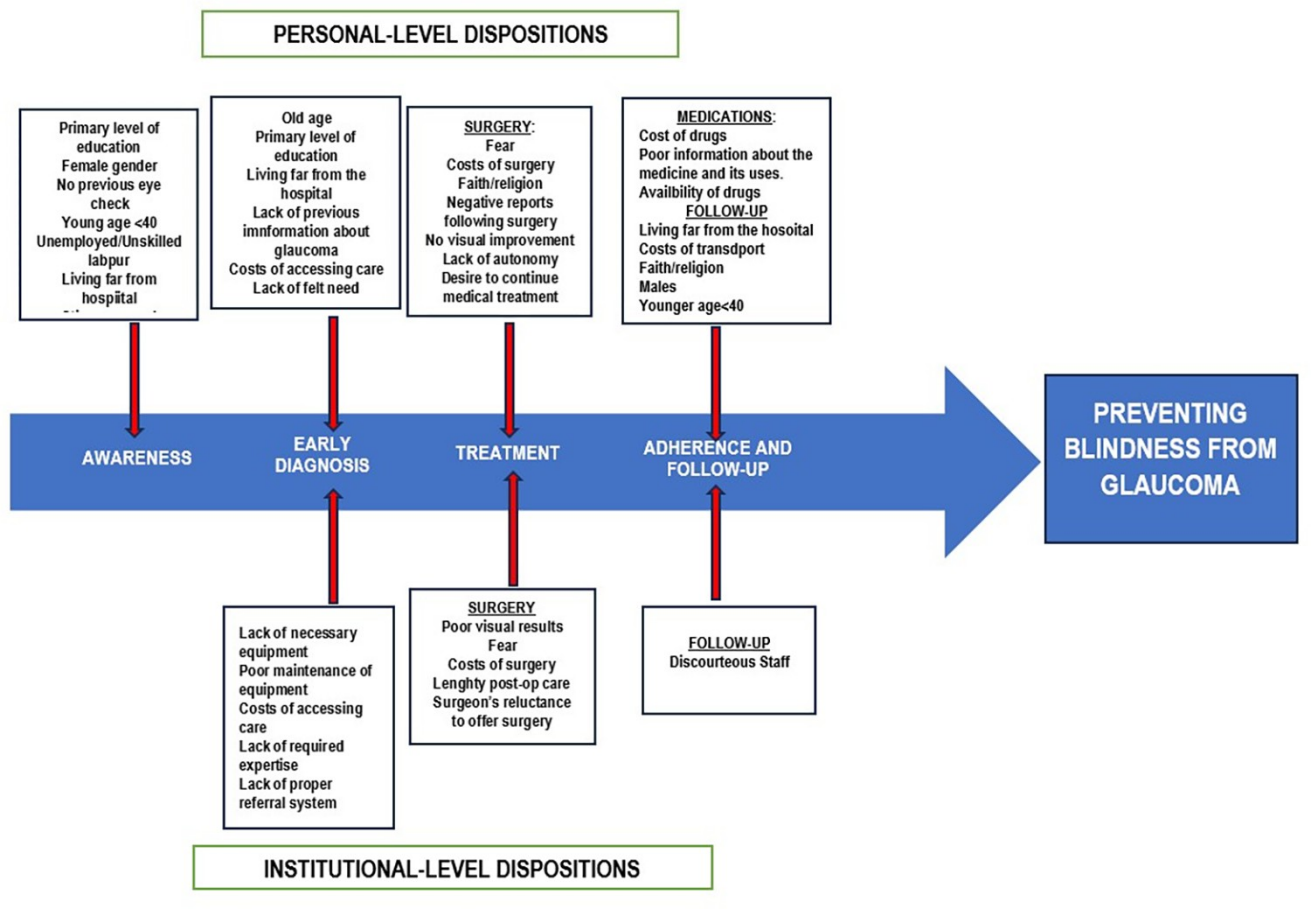
Factors limiting glaucoma care among glaucoma patients in Nigeria.

Fig 4 describes how these factors act along the glaucoma care pathway to limit optimum care and the achievement of “non-blindness”.

## Discussion

This scoping review set out to map the available evidence on the factors limiting glaucoma care among glaucoma patients in Nigeria and to determine how these factors impact the glaucoma care pathway. Personal-level dispositions limiting glaucoma care were low levels of education, unemployment, gender, living distance from the hospital, cost of care, and faith/religion. In contrast, institutional-level dispositions limiting glaucoma care included the lack of proper equipment and expertise needed to diagnose or manage glaucoma. These factors limiting glaucoma care in Nigeria may act alone or with other elements to restrict entry into the glaucoma care pathway (Fig 4).

Awareness or knowledge of glaucoma is essential to getting into the glaucoma care pathway. Poor education prevents access to glaucoma care by limiting the knowledge or understanding of glaucoma and the ability to get an early diagnosis [34, 35]. Furthermore, low levels of education are directly correlated with employment, which determines the ability of patients to afford care [36]. The cost of care for glaucoma is relatively high, estimated to cost about 394 USD for medical treatment and 283 USD for surgical care in Africa [37, 38]. The ability to afford care is an essential factor in accessing glaucoma services, and cost was a recurring factor limiting access to glaucoma services in this review. A combination of low levels of education, unemployment, or unskilled labour potentiates low levels of glaucoma awareness and knowledge, leading to blindness from glaucoma [39].

The personal-level dispositions/social determinants of blindness limiting glaucoma care in Nigeria mirror those described in other regional and African countries [40, 41]. These social determinants, which act at the individual level, like living environments, transport mobility, and social support to determine access to health care, are aptly captured by Pechansky’s dimension of availability and accommodation [42]. Living farther from the hospital increases the patient’s geographical inaccessibility to glaucoma information, diagnosis, and care, potentiating their inability to adhere to follow-up visits or purchase medications [43, 44]. While patients’ residence may not be modifiable, providing Primary healthcare-driven glaucoma services may serve as an answer to providing glaucoma services to people far away from secondary or tertiary hospitals [44].

Penchansky describes the dimension of acceptability as the factors which promote the utilisation of a service by actively seeking the service or product out [42]. In health systems, this is driven by the social determinants of health, like personal and social values, culture, gender, and patient autonomy [45]. These factors limit access to diagnosis, surgical uptake, and follow-up in accessing glaucoma care. The female gender does not have the economic, social, and cultural autonomy to make health-related decisions, which either delay health-seeking or prevent access to health care entirely [45]. Culture or religion may influence health-seeking behaviour or the response to health instructions regarding medication use or uptake of surgery.

Sub-saharan Africa has only 2.9 surgeons per million population [46], and Nigeria had only five glaucoma specialists until 2015 [38], while another survey in Botswana in 2015 showed the availability of only two Ophthalmologists [47]. In government hospitals, eye care equipment is either broken down or unavailable [48]. The dearth of qualified personnel and the lack of the necessary equipment required to diagnose glaucoma means that patients who may have benefitted from an early diagnosis end up slipping through the cracks of the glaucoma care pathway. Furthermore, the patient’s dissatisfaction from not getting the best service may prevent or deter the patient’s return in the future. The patient may also inform the community about the non-existence of proper hospital equipment or poor service, leading to poor utilisation and uptake of services. The same holds for discourteous hospital staff. Poor behaviour of hospital staff towards patients does not instil confidence or create an atmosphere of trust, which is vital in managing glaucoma and other eye conditions.

The factors limiting access to glaucoma care do not act in isolation. They work with other elements to affect the care of glaucoma across the glaucoma care pathway. Policies to provide financial protection for people seeking glaucoma care, improve education services, and provide eye care information in Primary healthcare or community health sessions are necessary to break this cycle leading to preventable blindness from glaucoma. In addition, better funding for eye care, training and retraining of eye care workers and better staff disposition to clients and equipment are needed to reduce or eliminate the institutional-level dispositions limiting glaucoma care in Nigeria.

### Evidence gaps/Limitations

While there were many reports in this review regarding the factors limiting glaucoma awareness, diagnosis, surgical treatment, follow-up, and adherence to medications by patients, there were no reports on factors limiting the medical treatment of glaucoma among patients with glaucoma. Furthermore, there were no reports regarding institutional-level dispositions limiting or influencing medication adherence during glaucoma care. Generally, there were few papers regarding institutional factors compared to patient factors. More evidence is needed regarding the contribution of institutional factors to limiting access to glaucoma care, especially regarding the medical treatment of glaucoma. Furthermore, mixed-method approaches to determining evidence on glaucoma care are recommended.

## Conclusion

The factors limiting glaucoma care in Nigeria prevent patients from accessing the needed care, ranging from personal factors like faith and culture to systemic factors like gender inequality, poor education, unemployment, and geographic inaccessibility. While most of these factors may be amenable to policy, a bottom-up approach is needed to improve the community’s awareness and uptake of glaucoma services. A shift from the over-dependence and reliance on tertiary hospitals, which are often far away from the people who need them, is required to bridge the information and service gap currently being witnessed. Furthermore, training and retraining of ophthalmologists in providing high-quality glaucoma care is vital if Nigeria is going to reduce the number of people going blind from glaucoma. In addition, it is crucial to develop a structural approach to providing high-quality, easy-to-understand information on glaucoma and eye health. Finally, more evidence needs to be generated regarding the contribution of our institutions and eye care workers to preventing glaucoma services.

## Supporting information

S1 Fig Search strategy. (PDF)

S2 Fig Data extraction instrument for observational studies. (PDF)

S3 Fig Data extraction instrument for qualitative studies. (PDF)

## References

[1] Tham Y-C, Li X, Wong TY, Quigley HA, Aung T, Cheng C-Y. Global prevalence of glaucoma and projections of glaucoma burden through 2040: a systematic review and meta-analysis. Ophthalmology. 2014;121(11):2081–90. doi: 10.1016/j.ophtha.2014.05.013 24974815

[2] Sun Y, Chen A, Zou M, Zhang Y, Jin L, Li Y, et al. Time trends, associations and prevalence of blindness and vision loss due to glaucoma: an analysis of observational data from the Global Burden of Disease Study 2017. BMJ Open. 2022;12(1):e053805. doi: 10.1136/bmjopen-2021-053805 34992115 PMC8739070

[3] Abdull MM, Gilbert CC, Evans J. Primary open angle glaucoma in northern Nigeria: stage at presentation and acceptance of treatment. BMC Ophthalmol. 2015 Aug 22;15:111. doi: 10.1186/s12886-015-0097-9 26296993 PMC4546340

[4] Lawan A. Pattern of presentation and outcome of surgical management of primary open angle glaucoma in Kano, Northern Nigeria. Ann Afr Med. 2007 Dec;6(4):180–5. doi: 10.4103/1596-3519.55700 18354943

[5] Omoti AE, Osahon AI, Waziri-Erameh MJM. Pattern of presentation of primary open-angle glaucoma in Benin City, Nigeria. Trop Doct. 2006 Apr;36(2):97–100. doi: 10.1258/004947506776593323 16611443

[6] Quigley HA, West SK, Rodriguez J, Munoz B, Klein R, Snyder R. The prevalence of glaucoma in a population-based study of Hispanic subjects: Proyecto VER. Arch Ophthalmol. 2001 Dec;119(12):1819–26. doi: 10.1001/archopht.119.12.1819 11735794

[7] Abdull MM, Sivasubramaniam S, Murthy GV, Gilbert C, Abubakar T, Ezelum C, et al. Causes of blindness and visual impairment in Nigeria: the Nigeria national blindness and visual impairment survey. Invest Ophthalmol Vis Sci. 2009 Sep;50(9):4114–20. doi: 10.1167/iovs.09-3507 19387071

[8] World Health Organisation. Strategies for the Prevention of Blindness in National Programmes: A Primary Health Care Approach. World Health Organization; 1997. 85–88 p.

[9] Thomas R. Glaucoma in developing countries. Indian J Ophthalmol [Internet]. 2012 [cited 2023 Sep 22];60(5):446–50. doi: 10.4103/0301-4738.100546 22944757 PMC3491273

[10] Kyari F, Adekoya B, Abdull MM, Mohammed AS, Garba F. The Current Status of Glaucoma and Glaucoma Care in Sub-Saharan Africa. Asia Pac J Ophthalmol (Phila). 2018 Dec;7(6):375–86. doi: 10.22608/APO.2018392 30574693

[11] Kyari F, Chandler CI, Martin M, Gilbert CE. So let me find my way, whatever it will cost me, rather than leaving myself in darkness: experiences of glaucoma in Nigeria. Glob Health Action. 2016;9:31886. doi: 10.3402/gha.v9.31886 27924740 PMC5141371

[12] Dineen BP, Gilbert CE, Rabiu MM, Kyari F, Mahdi AM, Abubakar T, et al. The Nigerian national blindness and visual impairment survey: Rationale, objectives and detailed methodology. BMC Ophthalmology. 2008;8:17–17. doi: 10.1186/1471-2415-8-17 18808712 PMC2572038

[13] Musa I, Bansal S, Kaleem MA. Barriers to Care in the Treatment of Glaucoma: Socioeconomic Elements That Impact the Diagnosis, Treatment, and Outcomes in Glaucoma Patients. Current Ophthalmology Reports [Internet]. 2022 Sep 1;10(3):85–90. doi: 10.1007/s40135-022-00292-6 35911786 PMC9325663

[14] Ng WS, Agarwal PK, Sidiki S, McKay L, Townend J, Azuara-Blanco A. The effect of socio-economic deprivation on severity of glaucoma at presentation. Br J Ophthalmol. 2010 Jan;94(1):85–7. doi: 10.1136/bjo.2008.153312 19628488

[15] Olawoye O, Fawole OI, Monye HI, Ashaye A. Eye Care Practices, Knowledge, and Attitude of Glaucoma Patients at Community Eye Outreach Screening in Nigeria. J West Afr Coll Surg. 2020 Dec;10(4):16–22. doi: 10.4103/jwas.jwas_48_22 35814968 PMC9267041

[16] Uche NJ, Udeh NN, Chuka-Okosa CM, Kizor-Akaraiwe NN, Uche EO. Glaucoma care and follow-up in sub-Saharan Africa: Is there a need for modification of counselling practices to improve awareness, knowledge and treatment acceptance profiles? A prospective cross-sectional study. Int Ophthalmol. 2020 Jun;40(6):1539–46. doi: 10.1007/s10792-020-01323-6 32088903

[17] Aghaji A, Gilbert C. Policies for primary eye health care in Nigeria: a case study. Community Eye Health [Internet]. 2021 [cited 2023 Sep 21];34(113):82–3. 36033406 PMC9412120

[18] Aghaji A, Burchett HED, Oguego N, Hameed S, Gilbert C. Human resource and governance challenges in the delivery of primary eye care: a mixed methods feasibility study in Nigeria. BMC Health Serv Res [Internet]. 2021 Dec 10 [cited 2023 Sep 21];21:1321. doi: 10.1186/s12913-021-07362-8 34893081 PMC8662916

[19] Aghaji A, Burchett HED, Oguego N, Hameed S, Gilbert C. Primary health care facility readiness to implement primary eye care in Nigeria: equipment, infrastructure, service delivery and health management information systems. BMC Health Serv Res [Internet]. 2021 Dec 20 [cited 2023 Sep 21];21:1360. doi: 10.1186/s12913-021-07359-3 34930271 PMC8690487

[20] MDJ P, C G, P M, AC T, H K. Chapter 11: Scoping Reviews. In: Z M, E A, editors. JBI Manual for Evidence Synthesis [Internet]. Joanna Briggs Institute; [cited 2023 Jun 4]. (JBI 2020).

[21] Tricco AC, Lillie E, Zarin W, O’Brien KK, Colquhoun H, Levac D, et al. PRISMA Extension for Scoping Reviews (PRISMA-ScR): Checklist and Explanation. Ann Intern Med [Internet]. 2018 Oct 2 [cited 2023 Jun 2];169(7):467–73. doi: 10.7326/M18-0850 30178033

[22] Page MJ, McKenzie JE, Bossuyt PM, Boutron I, Hoffmann TC, Mulrow CD, et al. The PRISMA 2020 statement: an updated guideline for reporting systematic reviews. BMJ [Internet]. 2021 Mar 29;372:n71. doi: 10.1136/bmj.n71 33782057 PMC8005924

[23] Tricco AC, Lillie E, Zarin W, O’Brien K, Colquhoun H, Kastner M, et al. A scoping review on the conduct and reporting of scoping reviews. BMC Medical Research Methodology [Internet]. 2016 Feb 9 [cited 2023 Jul 18];16(1):15. doi: 10.1186/s12874-016-0116-4 26857112 PMC4746911

[24] Adekoya BJ, Adepoju FG, Moshood KF, Balarabe AH. Challenges in the management of glaucoma in a developing country; A qualitative study of providers perspectives. Niger J Med [Internet]. 2015;24(4):315–22. 27487608

[25] Kizor-Akaraiwe NN. Follow-up and adherence to glaucoma care by newly diagnosed glaucoma patients in enugu, nigeria. Ophthalmic Epidemiol. 2019 Apr;26(2):140–6. doi: 10.1080/09286586.2018.1555263 30521414

[26] Adekoya BJ, Akinsola FB, Balogun BG, Balogun MM, Ibidapo OO. Patient refusal of glaucoma surgery and associated factors in Lagos, Nigeria. Middle East Afr J Ophthalmol. 2013 Jun;20(2):168–73. doi: 10.4103/0974-9233.110612 23741137 PMC3669495

[27] Isawumi MA, Hassan MB, Akinwusi PO, Adebimpe OW, Asekun-Olarinmoye EO, Christopher AC, et al. Awareness of and Attitude towards glaucoma among an adult rural population of Osun State, Southwest Nigeria. Middle East Afr J Ophthalmol. 2014 Jun;21(2):165–9. doi: 10.4103/0974-9233.129769 24791109 PMC4005182

[28] Abdull MM, Chandler C, Gilbert C. Glaucoma, “the silent thief of sight”: patients’ perspectives and health seeking behaviour in Bauchi, northern Nigeria. BMC Ophthalmol. 2016 Apr 21;16:44. doi: 10.1186/s12886-016-0220-6 27102524 PMC4839108

[29] Olawoye OO, Fawole O, Ashaye AO, Chan VF, Azuara-Blanco A, Congdon N. Effectiveness of community outreach screening for glaucoma in improving equity and access to eye care in Nigeria. Br J Ophthalmol. 2023 Jan;107(1):30–6. doi: 10.1136/bjophthalmol-2021-319355 34362773

[30] Momoh RO, Bunce C, Oko-Oboh GA, Gilbert CE. Advanced glaucoma at presentation is associated with poor follow-up among glaucoma patients attending a tertiary eye facility in Southern Nigeria. Ophthalmic Epidemiol. 2018 Jun;25(3):266–72. doi: 10.1080/09286586.2018.1424345 29336690

[31] Ashaye AO, Adeoye AO. Characteristics of patients who dropout from a glaucoma clinic. J Glaucoma. 2008 May;17(3):227–32. doi: 10.1097/IJG.0b013e31815768b3 18414110

[32] Onwubiko SN, Udeh NN, Nkwegu O, Ukwu DO, Nwachukwu NZ. Glaucoma care in Nigeria: Is the current practice poised to tackle this emerging sight-threatening disease? Int Ophthalmol. 2019 Oct;39(10):2385–90. doi: 10.1007/s10792-019-01078-9 30710253

[33] Adekoya BJ, Adepoju FG, Moshood KF, Balarabe AH. CHALLENGES IN THE MANAGEMENT OF GLAUCOMA IN A DEVELOPING COUNTRY; A QUALITATIVE STUDY OF PROVIDERS’ PERSPECTIVES. Niger J Med [Internet]. 2015 [cited 10AD Jan 1];24(4):315–22. 27487608

[34] Murdoch C, Opoku K, Murdoch I. Awareness of Glaucoma and Eye Health Services Among Faith-based Communities in Kumasi, Ghana. J Glaucoma. 2016 Oct;25(10):e850–4. doi: 10.1097/IJG.0000000000000462 27300644

[35] De-Gaulle VF, Dako-Gyeke P. Glaucoma awareness, knowledge, perception of risk and eye screening behaviour among residents of Abokobi, Ghana. BMC Ophthalmol. 2016 Nov 17;16(1):204. doi: 10.1186/s12886-016-0376-0 27855682 PMC5114832

[36] Tafida A, Kyari F, Abdull MM, Sivasubramaniam S, Murthy GV, Kana I, et al. Poverty and Blindness in Nigeria: Results from the National Survey of Blindness and Visual Impairment. Ophthalmic Epidemiol. 2015;22(5):333–41. doi: 10.3109/09286586.2015.1077259 26395660

[37] Smith AF, Negretti G, Mascaro A, Bokre D, Baker H, Dhalla K, et al. Glaucoma Control Strategies in Sub-Saharan Africa: A Review of the Clinical and Health Economic Evidence. Ophthalmic Epidemiol. 2018;25(5–6):419–35. doi: 10.1080/09286586.2018.1501499 30059637

[38] Kizor-Akaraiwe NN, Olawoye O. Allocating resources for glaucoma care—A Review. 2019;

[39] Tamrat L, Gessesse GW, Gelaw Y. Adherence to topical glaucoma medications in Ethiopian patients. Middle East Afr J Ophthalmol. 2015;22(1):59–63. doi: 10.4103/0974-9233.148350 25624675 PMC4302478

[40] Tshivhase S, Khoza LB. Challenges Contributing to Loss to Follow-up as Experienced by Glaucoma Patients in the Vhembe District of Limpopo Province, South Africa. The Open Public Health Journal [Internet]. 2020 Oct 26 [cited 2023 Sep 23];13(1). 10.2174/1874944502013010531

[41] Damji KF, Nazarali S, Giorgis A, Kiage D, Marco S, Philippin H, et al. STOP Glaucoma in Sub Saharan Africa: enhancing awareness, detection, management, and capacity for glaucoma care. Expert Review of Ophthalmology [Internet]. 2017;12(3):197–206. doi: https%3A//doi.org/10.1080/17469899.2017.1295848

[42] Penchansky R, Thomas JW. The concept of access: definition and relationship to consumer satisfaction. Med Care. 1981 Feb;19(2):127–40. doi: 10.1097/00005650-198102000-00001 7206846

[43] Funk IT, Strelow BA, Klifto MR, Knight OJ, Buren EV, Lin F-C, et al. The Relationship of Travel Distance to Postoperative Follow-up Care on Glaucoma Surgery Outcomes. J Glaucoma [Internet]. 2020 Nov [cited 2023 Sep 23];29(11):1056–64. doi: 10.1097/IJG.0000000000001609 32694285 PMC7658019

[44] Delgado MF, Abdelrahman AM, Terahi M, Woll JJMQ, Gil-Carrasco F, Cook C, et al. Management Of Glaucoma In Developing Countries: Challenges And Opportunities For Improvement. CEOR [Internet]. 2019 Sep 27 [cited 2023 Sep 23];11:591–604. doi: 10.2147/CEOR.S218277 31632107 PMC6776288

[45] Azad AD, Charles AG, Ding Q, Trickey AW, Wren SM. The gender gap and healthcare: associations between gender roles and factors affecting healthcare access in Central Malawi, June–August 2017. Archives of Public Health [Internet]. 2020 Nov 17;78(1):119. doi: 10.1186/s13690-020-00497-w 33292511 PMC7672876

[46] Palmer JJ, Chinanayi F, Gilbert A, Pillay D, Fox S, Jaggernath J, et al. Mapping human resources for eye health in 21 countries of sub-Saharan Africa: current progress towards VISION 2020. Human Resources for Health [Internet]. 2014 Aug 15 [cited 2023 Sep 23];12(1):44. doi: 10.1186/1478-4491-12-44 25128163 PMC4237800

[47] Razai MS, Jackson DJ, Falama R, Mongwa M, Mutapanduwa MG, Baemisi C, et al. The Capacity of Eye Care Services for Patients with Glaucoma in Botswana. Ophthalmic Epidemiol. 2015;22(6):403–8. doi: 10.3109/09286586.2015.1010689 26196853

[48] Adekoya BJ, Shah SP, Adepoju FG. Managing glaucoma in Lagos State, Nigeria—availability of Human resources and equipment. Niger Postgrad Med J. 2013 Jun;20(2):111–5. 23959351

