# Supplementary material for: Factors limiting glaucoma care among glaucoma patients in Nigeria: A scoping review
Source: PLOS Glob Public Health. 2024 Jan 26;4(1):e0002488. doi: 10.1371/journal.pgph.0002488 (PMC10817109; doi:10.1371/journal.pgph.0002488)
Supplement: S1 Fig — (PDF) [file pgph.0002488.s001.pdf]

FACTORS OR REASONS OR BASIS OR BARRIERS OR OBSTACLES OR REFUSAL OR IMPEDIMENTS OR CHALLENGES

AND

ACCESS OR UTILISATION OR USE OR UPTAKE OR AWARENESS OR KNOWLEDGE OR UNDERSTANDING OR ACCEPTANCE OR ADHERENCE OR FOLLOW-UP OR COMPLIANCE

AND

GLAUCOMA OR 'GLAUCOMA CARE' OR 'GLAUCOMA DIAGNOSIS' OR 'GLAUCOMA TREATMENT' OR 'GLAUCOMA SURG*' OR TRABECULECTOMY OR LASER OR 'MEDICAL GLAUCOMA TREATMENT' OR 'SURGICAL GLAUCOMA TREATMENT'

AND

NIGERIA*
