# Supplementary material for: Factors limiting glaucoma care among glaucoma patients in Nigeria: A scoping review
Source: PLOS Glob Public Health. 2024 Jan 26;4(1):e0002488. doi: 10.1371/journal.pgph.0002488 (PMC10817109; doi:10.1371/journal.pgph.0002488)
Supplement: S2 Fig — (PDF) [file pgph.0002488.s002.pdf]

DATA EXTRACTION TOOL FOR OBSERVATIONAL STUDIES

REVIEWER:

DATE OF REVIEW:

AUTHOR: JOURNAL: YEAR:

STUDY METHOD:

- o PROSPECTIVE COHORT
- o RETROSPECTIVE COHORT
- o ANALYTICAL CROSS SECTIONAL
- o DESCRIPTIVE CROSS SECTIONAL

PARTICIPANTS:

A. SETTING
B. SAMPLE SIZE
C. POPULATIION
D. INTERVENTION

RESULTS

A. DEMOGRAPHICS

AGE	SEX	OCCUPATION	RESIDENCE

B. FACTORS AFFECTING

AWARENESS	
DIAGNOSIS	
TREATMENT	
ADJERENCE/FOLLOW-UP	

C. AUTHORS CONCLUSIONS
