# Supplementary material for: Factors limiting glaucoma care among glaucoma patients in Nigeria: A scoping review
Source: PLOS Glob Public Health. 2024 Jan 26;4(1):e0002488. doi: 10.1371/journal.pgph.0002488 (PMC10817109; doi:10.1371/journal.pgph.0002488)
Supplement: S3 Fig — (PDF) [file pgph.0002488.s003.pdf]

DATA EXTRACTION TOOL FOR INTERPRETIVE STUDIES

REVIEWER:

DATE OF REVIEW:

AUTHOR: JOURNAL: YEAR:

STUDY METHOD:

A. PARTICIPANTS:
B. SETTING
C. SAMPLE SIZE
D. PARTICIPANTS
E. DATA ANALYSIS

RESULTS

D. DEMOGRAPHICS

AGE	SEX	OCCUPATION	RESIDENCE

E. FACTORS AFFECTING

AWARENESS	
DIAGNOSIS	
TREATMENT	
ADJERENCE/FOLLOW-UP	

NB: REFERENCE THE PAGE, LINE OR PARAGRAPH STATING THE RESULT WITH THE RELEVANT ILLUSTRATION

F. AUTHORS CONCLUSIONS
